# Clinical Implications of Nadir Serum Prostate-Specific Antigen Levels After Transurethral Enucleation of the Prostate

**DOI:** 10.3389/fonc.2022.949275

**Published:** 2022-07-15

**Authors:** Yung-Ting Cheng, Jian-Hua Hong, Yu-Chuan Lu, Yi-Kai Chang, Shih-Chun Hung, Kuo-Kang Feng, Shih-Ping Liu, Po-Ming Chow, Hong-Chiang Chang, Chung-Hsin Chen, Yeong-Shiau Pu

**Affiliations:** ^1^ Department of Urology, National Taiwan University Hospital, Taipei, Taiwan; ^2^ Department of Urology, National Taiwan University Hospital Hsin-Chu Branch, Hsinchu City, Taiwan; ^3^ Department of Urology, National Taiwan University Cancer Center, Taipei, Taiwan

**Keywords:** benign prostatic hyperplasia, nadir, prediction, prostate cancer, PSA kinetics

## Abstract

**Objective:**

Prostate-specific antigen levels after transurethral enucleation of the prostate may serve as indicators of residual cancer foci. The objective of this study was to investigate the association between the post-transurethral enucleation of the prostate nadir prostate-specific antigen level and prostate cancer.

**Materials and Methods:**

We retrospectively reviewed the data of 428 men who underwent transurethral enucleation of the prostate between March 2015 and April 2021. Based on the following exclusion criteria, we excluded 106 men from our analysis: men with metastatic prostate cancer, incomplete transurethral enucleation of the prostate, and missing prostate-specific antigen or prostate size data. Three hundred and twenty-two patients were finally enrolled in our study. These patients were classified into four groups according to the surgical pathology: benign, transition zone (cancer only in the adenoma or transition zone), peripheral zone, and transition and peripheral zones. The optimal cutoff post-transurethral enucleation of the prostate nadir prostate-specific antigen level that predicted residual prostate cancer was determined using receiver operating characteristic curve analysis.

**Results:**

In total, 71 (22.0%) men exhibited prostate cancer (median follow-up, 38.0 months). The benign and combined cancer groups showed similar adenoma removal rates (103.0% and 106.7%, respectively). The median nadir prostate-specific antigen levels after transurethral enucleation of the prostate were 0.76, 0.63, 1.79, and 1.70 ng/ml in the benign, transition zone, peripheral zone, and transition and peripheral zone groups, respectively (p < 0.001), with no difference between the benign and transition zone groups (p = 0.458); this suggested that complete transurethral enucleation of the prostate removed all cancer nests in the adenoma in the transition zone group. Receiver operating characteristic curve analysis showed that nadir prostate-specific antigen ≧1.7 ng/ml predicted residual cancer (area under the curve: 0.787) or cancer with a Gleason score of ≧7 (area under the curve: 0.816) in the remaining prostate. Limitations include the retrospective design and the perioperative peripheral zone biopsy rate.

**Conclusions:**

The post-transurethral enucleation of the prostate nadir prostate-specific antigen ≧1.7 ng/ml after complete transurethral enucleation of the prostate can predict significant residual cancer. Prostate cancer patients with low post-transurethral enucleation of the prostate prostate-specific antigen levels can be conservatively managed.

## Introduction

Benign prostatic hyperplasia (BPH) is a common cause of urinary symptoms in older men. The prostate can be anatomically divided into the transition zone (TZ), central zone (CZ), peripheral zone (PZ), and anterior fibromuscular stroma (1). An enlarged TZ, also known as adenoma, is considered the major component of BPH (1).

Transurethral resection of the prostate (TURP) is the gold standard surgical treatment for BPH. Transurethral enucleation of the prostate (TUEP) is a new technique for more complete adenoma removal by dissection along the surgical plane between TZ and PZ/CZ (2). The surgical cases of TUEP had tripled from 2008 to 2014 (3). It removes prostate adenomas almost completely, similar to open simple prostatectomy (4–7). With complete TUEP, only PZ, CZ, and anterior fibromuscular stroma of the prostate are left behind after surgery (8).

Serum prostate-specific antigen (PSA) levels can be affected by age, race, prostate volume, and prostate diseases (9–11). The “reset” PSA level after radical prostatectomy for prostate cancer (PCa) has been used as a marker of surgical completeness, presence of residual PCa, and postoperative follow-up (12). The nadir PSA level after TURP is theoretically produced from the remaining prostate tissue, including PZ, CZ, and residual TZ, if any. Compared with TURP, TUEP can remove the adenoma more completely and may generate a new nadir PSA secreted only from PZ+CZ (13, 14). The nadir PSA level after complete TUEP may serve as a new indicator for not only the completeness of TUEP but also the secretory activity in the remaining prostate.

We hypothesized that the nadir level of PSA secreted from the remaining PZ and CZ after TUEP remains stable and that the nadir and follow-up PSA levels may serve as biomarkers of residual and/or recurrent PCa (15, 16). Accordingly, the aim of this study was to investigate the oncological implications of the post-TUEP nadir PSA level and determine its association with PCa.

## Materials and Methods

### Patient Cohort and Risk Grouping

**Figure 1:**
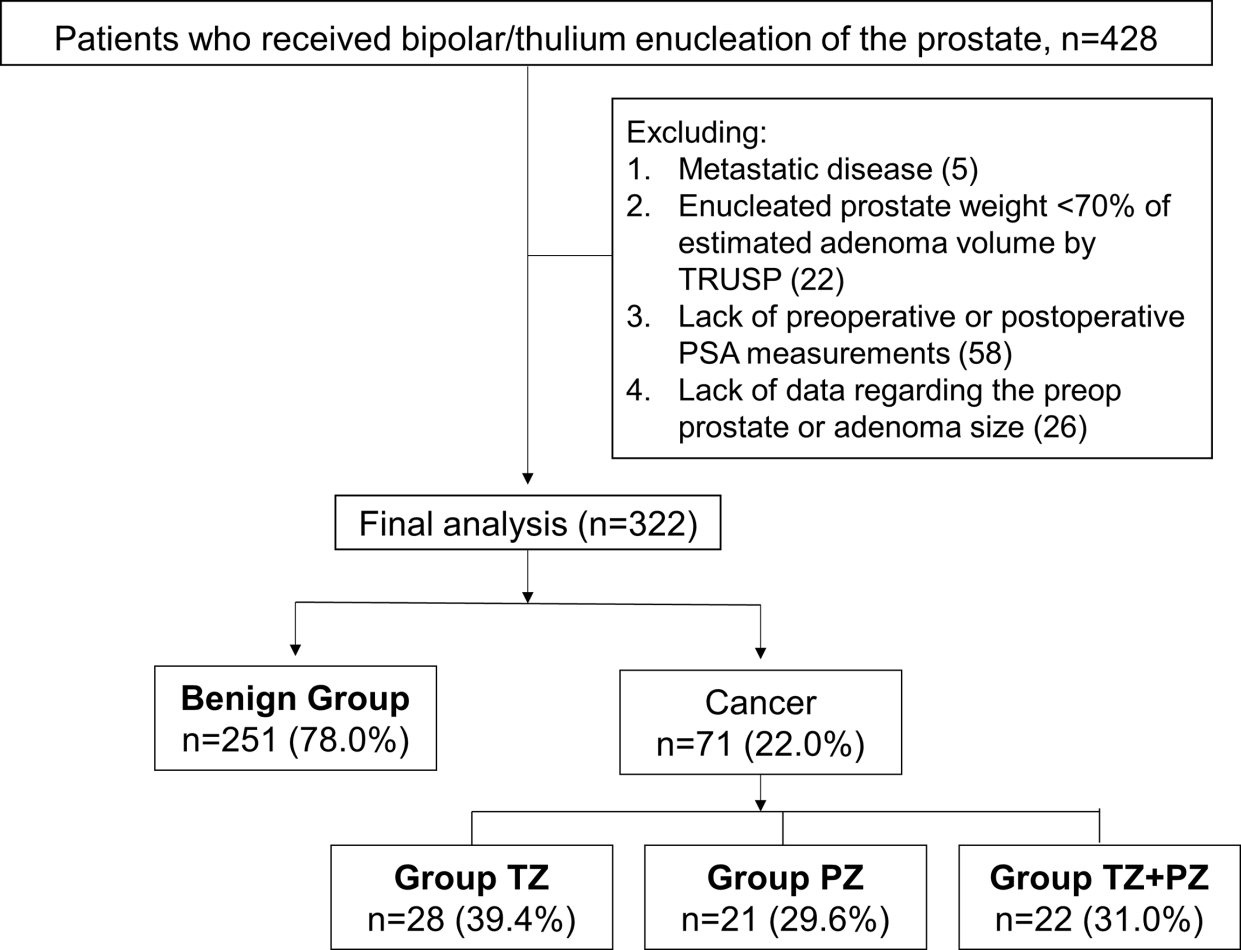
Patient cohort based on the surgical pathology for men who underwent transurethral prostate enucleation. Preop, preoperative; postop, postoperative; PSA, prostate-specific antigen; PZ, peripheral zone; TRUSP, transrectal ultrasound of the prostate; TZ, transition zone Some men were excluded because of the presence of more than one exclusion criterion.

Data for a cohort of 428 patients who underwent bipolar or thulium laser TUEP between March 2015 and April 2021 were retrospectively reviewed. This study was approved by the Research Ethics Committee Office of National Taiwan University Hospital (202009014RINA). The need for informed consent was waived because of the retrospective nature of the study. The exclusion criteria were as follows (1): metastatic PCa (2), enucleated prostate weight <70% of the estimated adenoma volume measured by transrectal ultrasound of the prostate (TRUSP) (3), missing pre- or postoperative PSA data, and (4) lack of data regarding the preoperative prostate or adenoma size. After exclusion of 106 men who were ineligible for analysis, 322 remained in the final analysis (**Figure 1**). The patients were divided into benign and cancer groups according to the results of surgical pathology with or without TRUSP-guided biopsy (TRUSP Bx). Based on the zonal distribution of the detected cancer, patients were further divided into TZ (cancer found only in TZ), PZ (cancer found only in PZ using TRUSP Bx, with no cancer found in the adenoma during TUEP), and TZ and PZ (cancer found in both TZ and PZ) groups (**Figure 1**).

### PSA and TRUSP

Prostate and adenoma sizes determined by TRUSP were reviewed and recalculated by the same urologist (Y. T. Cheng) using the caliper-based method: width × length × height × π/6 (17). 5α-reductase inhibitors were preoperatively prescribed to 76 (23.6%) of the 322 men and discontinued after TUEP. The median duration of 5αRI use was 9 months (interquartile range [IQR]: 3–28.5 months). We restored the preoperative PSA level by doubling the PSA values in these patients, as previously described (18). For patients diagnosed with PCa, PSA after definitive cancer treatment (such as radiotherapy, radical prostatectomy, or hormone therapy) will not be analyzed.

### Perioperative Parameters and Endpoints

The use of either bipolar, laser, or combined surgical techniques was in accordance with shared decisions made by surgeons and patients. Bipolar TUEP was performed using the PK technology system ESG-400 (Olympus Winter & Ibe GmbH, Hamburg, Germany) with power settings of 230 and 160 W for cutting and coagulation, respectively. Thulium laser TUEP was performed using the 120-W vela XL laser device (Boston Scientific Co., MA, USA). Percentage adenoma removal was defined as follows: [enucleated prostate weight (g)/adenoma size (ml)] × 100%. PSA levels were checked before and within 12 weeks of TUEP and every 3 to 6 months thereafter. Percentage PSA reduction was defined as follows: [(preoperative PSA − postoperative nadir PSA)/preoperative PSA] × 100%. PSA reduction per gram of enucleated prostate weight (PSAW) was defined as (preoperative PSA − postoperative nadir PSA)/enucleated prostate weight (g). The study endpoint was the cutoff nadir PSA level for predicting residual cancer in the remaining prostate after TUEP.

### Statistical Analysis

All data were analyzed using SPSS version 20 (IBM Corp, Inc., Chicago, IL, USA) and GraphPad Prism (GraphPad Software, Inc., San Diego, CA, USA) version 5.01.336. Non-parametric variables are presented as medians and 95% confidence intervals (95% CIs). The Mann–Whitney U and Kruskal−Wallis tests were used to compare non-parametric continuous variables. The optimal cutoff post-TUEP nadir PSA level for the prediction of residual cancer in the remaining prostate (cancer in PZ) was determined using receiver operating characteristic curve analysis (ROC-CA). The median time to PSA progression (TTPP) (defined as PSA≥1.7 ng/ml) was calculated using the Kaplan–Meier method. The log-rank test was used to compare survival between groups. All tests were two-sided, and p < 0.05 was considered statistically significant.

## Results

### Patient Characteristics and Preoperative Parameters

The median duration from TUEP to the final analysis (February 22, 2022) was 38.0 months (IQR: 17.0–52.0). The median duration to the last follow-up or censor date was 18.5 months (IQR: 5.0–32.0). Of the 322 men, 71 (22.0%) were diagnosed with PCa using TUEP and/or TRUSP Bx. There were no significant differences in preoperative prostate or adenoma sizes between the benign and combined cancer groups (**Table 1**).

**Table 1:** Characteristics and perioperative parameters of patients who underwent TUEP.

	Benign group(n = 251)	Cancer group(n = 71)				
		All cancer groupsn = 71	All cancer group vs. benign group	Group TZ^※^n = 28	Group PZn = 21	Group TZ+PZn = 22
	Median (95% CI)	Median (95% CI)	P-value	Median (95% CI)	Median (95% CI)	Median (95% CI)
Age, years	70.0 (69.0, 71.0)	74.0 (70.9, 74.0)	**0.009**	76.0 (70.7, 76.7)	72.0 (68.5, 73.2)	74.0 (69.8, 74.9)
Preoperative parameters						
Prostate size, ml	75.0 (75.5, 84.3)	65.0 (66.7, 83.3)	0.244	54.5 (55.4, 83.4)	83 (71.7, 102.8)	66.0 (55.6, 85.4)
Adenoma size, ml	42.0 (44.1, 51.2)	37.0 (36.3, 48.8)	0.165	25.0 (26.0, 46.6)	45.0 (41.9, 66.0)	34.5 (29.2, 50.3)
PSA, ng/ml	7.0 (7.9, 10.0)	13.0 (16.7, 29.6)	**<0.001**	11.0 (9.1, 29.2)	10.0 (8.8, 31.0)	25.0 (17.7, 44.9)
PSAD, ng/ml^2^	0.09 (0.10, 0.13)	0.18 (0.25, 0.42)	**<0.001**	0.17 (0.15, 0.43)	0.14 (0.11, 0.35)	0.33 (0.31, 0.66)
Intraoperative parameters						
Enucleated prostate weight, gm	49.0 (48.9, 57.5)	40.0 (38.3, 54.6)	0.080	36.5 (28.2, 63.5)	52.0 (43.5, 65.3)	23.0 (28.3, 51.1)
Operation time, min	93.0 (90.9, 101.2)	90.0 (88.4, 106.8)	0.697	83.0 (73.5, 103.8)	105.0 (93.0, 125.3)	91.0 (79.8, 115.9)
Percentage adenoma removal, %	103.0 (110.5, 122.6)	106.7 (105.8, 128.2)	0.720	122.3 (110.7, 159.9)	103.5 (93.8, 118.5)	103.7 (91.4, 116.7)
Gleason score,						
6		26 (36.6%)		12 (42.9%)	10 (47.6%)	4 (18.2%)
7		30 (42.3%)		13 (46.4%)	7 (33.3%)	10 (45.5%)
8–10		15 (21.1%)		3 (10.7%)	4 (19.0%)	8 (36.4%)
Postoperative parameters						
PSA nadir, ng/ml	0.76 (0.6, 1.1)	1.0 (1.4, 6.9)	**<0.001**	0.63 (0.5, 1.6)	1.79 (0, 10.1)^‡^	1.70 (0, 15.0)^§^
Time to PSA nadir, weeks	12.4 (25.1, 34.7)	10.3 (16.0, 37.6)	0.165	12.1 (11.7, 59.8)	12.6 (12.3, 39.2)	8.4 (2.2, 30.4)
Percentage PSA reduction, %	91.1 (85.2, 88.6)	90.5 (77.8, 88.0)	0.915	94.1 (81.9, 93.8)	85.7 (61.4, 86.9)	90.9 (77.0, 93.5)
PSA velocity, ng/ml/year	0.2 (0.4, 11)	0.8 (1.6, 6.4)	**<0.001**	0.19 (0, 1.9)^†^	0.9 (0.1, 10.7)	3.3 (0.60, 10.4)

TUEP, transurethral enucleation of the prostate; CI, confidence interval; PSA, prostate-specific antigen; PSAD, PSA density; PSA reduction percentage, (preoperative PSA − postoperative nadir PSA)/preoperative PSA; PZ, peripheral zone; enucleated adenoma percentage, enucleated prostate weight/adenoma size on TRUS; TZ, transition zone; TRUSP, transrectal ultrasound of the prostate.

**^※^** Fifteen out of 28 (50%) patients in the TZ group underwent perioperative TRUSP-guided biopsy.

^†^The lower limit of the confidence interval was −0.16 but is presented as 0 because there was no negative PSA value.

^‡^The lower limit of the confidence interval was −0.07 but is presented as 0 because there was no negative nadir PSA value.

^§^The lower limit of the confidence interval was −0.68 but is presented as 0 because there was no negative PSA value.

P-value < 0.05 was considered statistically signiﬁcant and provided as bold values.

Of the 322 men, 248 (77.0%) had a PSA level of >4 ng/ml or abnormal digital rectal examination before TUEP. TRUSP Bx (≥12 cores) was performed for 157 (63.3%) patients; 93 (59.2%) underwent TRUSP Bx concurrently with TUEP while 29 (18.5%) and 35 (22.3%) underwent the procedure before and after TUEP, respectively. The remaining 91 (36.7%) men with elevated PSA did not undergo TRUSP Bx because of the low likelihood of cancer or refusal by the patients. Of note, 97/251 (38.6%) patients with benign tumors and 60/71 (84.5%) patients in the combined cancer group had undergone TRUSP Bx at least once.

### Postoperative Parameters and PSA Reduction

**Figure 2:**
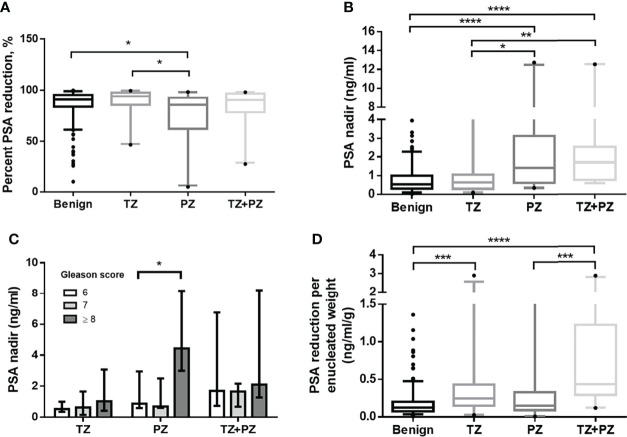
Postoperative PSA parameters for men treated by transurethral prostate enucleation. **(A)** Percentage PSA reduction by patient groups (p = 0.004, Kruskal–Wallis test) **(B)** Nadir PSA values by patient groups (p < 0.001, Kruskal–Wallis test) **(C)** Nadir PSA values by the Gleason score in each cancer group **(D)** PSA reduction per enucleated prostate weight by patient groups (p < 0.001, Kruskal–Wallis test) PSA, prostate-specific antigen; TZ, transition zone; PZ, peripheral zone *p < 0.05, **p < 0.01, ***p < 0.001, ****p < 0.0001, Mann–Whitney U test.

The benign and combined cancer groups had similar adenoma removal rates (median 103.0% and 106.7%, respectively, p = 0.72) and high percentage PSA reduction rates (median 91.1% vs. 90.5%, respectively, p = 0.92). The percentage PSA reduction was significantly higher in the benign and TZ groups (p = 0.04 and p = 0.02, respectively) than in the PZ group; this suggested that high PSA-secreting cancer nests may have been removed in group TZ but left behind in group PZ after TUEP (**Table 1** and **Figure 2A**).

The nadir PSA was significantly lower in the benign group than in the combined cancer group (**Table 1**). Interestingly, there was no significant difference in the nadir PSA between the benign and TZ groups (median 0.76 vs. 0.63, respectively, p = 0.458), whereas it was significantly lower in the TZ group than in the PZ (p = 0.02) and TZ+PZ (p = 0.002) groups (**Figure 2B**). This suggested that cancer nests in the TZ group were efficiently removed by TUEP and that the nadir PSA was lower in the TZ group as the benign group and higher in the PZ or TZ+PZ group, where residual cancer nests were present after TUEP.

The median Gleason score (GS) was 7 in the TZ, PZ, and TZ+PZ groups (**Table 1**). Patients with GS ≥8 had significantly higher nadir PSA than did those with GS 7 or 6 in the PZ group (GS ≥8 vs. GS 7, 4.4 vs. 0.7 ng/ml, p = 0.04; GS ≥8 vs. GS 6, 4.4 vs. 0.9 ng/ml, p = 0.04, respectively), but not in the TZ group (GS ≥8, 7, and 6, 1.0, 0.6, and 0.5 ng/ml, respectively, p = 0.364, Kruskal–Wallis test) (**Figure 2C**). This suggested that if TZ tumors were completely removed by TUEP, GS of the removed adenoma would not influence the nadir PSA.

### Optimal Nadir PSA Cutoff

**Figure 3:**
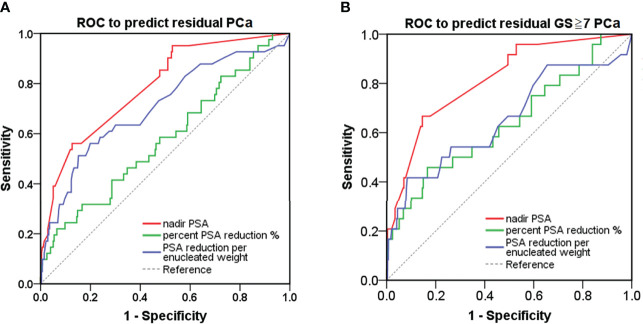
ROC curve analysis for detection of PCa in the remaining prostate after transurethral prostate enucleation. ROC curve analysis of postoperative PSA parameters to predict **(A)** residual PCa or **(B)** residual PCa with GS ≥7 in the remaining prostate after transurethral enucleation of the prostate. GS, Gleason score; PSA, prostate-specific antigen; PCa, prostate cancer; ROC, receiver operating characteristic.

ROC-CA showed that the nadir PSA, compared with other predictors, had the highest area under the curve (AUC) for predicting residual PCa and residual cancer nests with GS ≥7 in the remaining prostate (AUC: 0.787 and 0.816, respectively, both p < 0.001; **Figures 3A, B**, and **Table 2**). The Youden index method showed that the optimal cutoff nadir PSA level that predicted residual cancer and GS ≥7 cancer was 1.7 ng/ml (**Table 2**). Up to 90.0%, 78.6%, 47.6%, and 45.5% men in the benign, TZ, PZ, and TZ+PZ groups, respectively, had nadir PSA <1.7 ng/ml.

**Table 2:** Prediction models: all prostate cancers or prostate cancers with GS ≥7 using post-TUEP PSA derivatives.

Predictors	PSA nadir (ng/ml)		Percentage PSA reduction (%)		PSAW (ng/ml/g)	
Predicting outcome	All PCa	PCa with GS ≥7	All PCa	PCa with GS ≥7	All PCa	PCa with GS ≥7
AUC (95% CI)	0.787 (0.714–0.861)	0.816 (0.728–0.903)	0.577 (0.479–0.675)	0.640 (0.514–0.761)	0.706 (0.613–0.799)	0.655 (0.523–0.787)
P-value	**<0.001**	**<0.001**	0.111	0.022	**<0.001**	**0.012**
Optimal cutoff	1.687	1.687	90.894	79.879	0.265	0.430
Youden’s index	0.434	0.523	0.107	0.295	0.362	0.335
Sensitivity at threshold	0.561	0.667	0.585	0.458	0.561	0.417
Specificity at threshold	0.873	0.857	0.522	0.836	0.801	0.918

AUC, area under the receiver operating characteristic curve; CI, confidence interval; GS, Gleason score; PCa, prostate cancer; PSA, prostate-specific antigen; PSAW, PSA reduction per enucleated weight; TUEP, transurethral enucleation of the prostate.

P-value < 0.05 was considered statistically signiﬁcant and provided as bold values.

### PSAW

PSAW was significantly lower in the benign group (0.13 ng/ml/g) than in the TZ (0.24 ng/ml/g) and TZ+PZ (0.44 ng/ml/g) groups (both p < 0.001), but not the PZ group (0.15 ng/ml/g, p = 0.234, **Figure 2D**), possibly because most cancer nests remained in the PZ group, resulting in low postoperative percentage PSA reduction. This also explains the higher post-TUEP nadir PSA in the PZ and TZ+PZ groups than in the benign and TZ groups. PSAW had a fair AUC for predicting all cancers and GS ≥7 cancer in the remaining prostate at cutoff values of 0.265 and 0.430 ng/ml/g, with AUCs of 0.706 and 0.655 (p < 0.001 and p = 0.01), respectively (**Table 2**).

### Postoperative Follow-up and PSA progression

**Figure 4:**
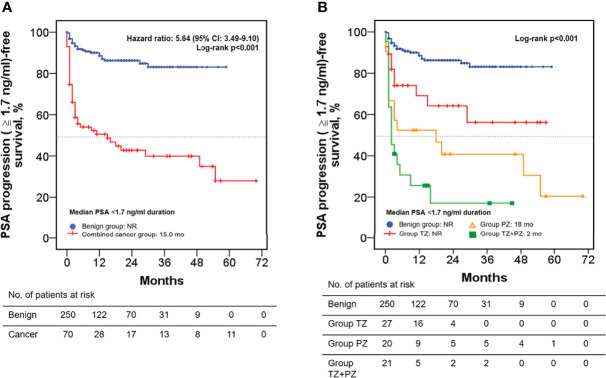
Kaplan–Meier analysis of PSA progression-free survival in men treated by transurethral prostate enucleation. CI, confidence interval; mo, months; NR, not reached; PSA, prostate-specific antigen; PZ, peripheral zone; TZ, transition zone.

The median TTPP (≥1.7 ng/ml) was not reached (>60 months) in the benign group, whereas it was only 15 months in the combined cancer group (hazard ratio: 5.64; 95% CI, 3.49–9.10; log-rank test: p < 0.001; **Figure 4A**). The median TTPP was also not reached in the TZ group, but it was 18 and 2 months in the PZ and TZ+PZ groups, respectively (log-rank test: p < 0.001) (**Figure 4B**). The postoperative PSA velocity (PSAV) was low in the benign and TZ groups and high in the PZ and TZ+PZ groups (**Table 1**); PSAV in the benign group was similar to that in the TZ group (p = 0.767) and significantly higher than that in the PZ and TZ+PZ groups (both p < 0.05), indicating the significant existence of residual cancer in the PZ and TZ+PZ groups.

Of the 71 patients with PC, subsequent treatment taken included 38 (53.5%) patients with active surveillance, 10 (14.1%) with watchful waiting, 18 (25.4%) with definitive radiotherapy, and 5 (7.0%) with radical prostatectomy. None of the 71 men died of PCa.

## Discussion

This study has several important clinical implications. First, after complete TUEP, nadir PSA ≥1.7 ng/ml may inform the presence of residual PCa nests and/or foci with GS ≥7 in the remaining prostate. In contrast, men with nadir PSA <1.7 after TUEP may have no significant PCa foci in the remaining prostate. Second, for men with *de novo* PCa found in the removed adenoma, active surveillance may be preferred overactive treatments if the nadir PSA is <1.7. Third, the basal PSA level secreted from the remaining benign prostate (PZ/CZ) seems to be stable and lower than the new threshold of 1.7 ng/ml. Fourth, for men with initial nadir PSA <1.7 ng/ml after TUEP and progressive PSA elevation to ≥1.7 ng/ml during follow-up, cancer foci or GS ≥7 cancer should be suspected and re-biopsy of the remaining prostate should be considered, which however needs a longer follow-up and more data to confirm. Traditionally, the nadir PSA after successful radical prostatectomy for PCa is undetectable or <0.1 ng/ml. Similarly, the 1.7-ng/ml threshold may be considered a new baseline for follow-up after complete TUEP.

Previous reports showed that PCa may be incidentally found in 5.4%–12% surgical specimens from TURP, most of which are considered indolent or clinically insignificant (19–21). PSA-derived markers after prostate enucleation can be used to differentiate BPH from incidental PCa and monitor possible cancer progression (22). Favorable oncological outcomes in men with incidental PCa detected during TUEP have made active surveillance an attractive option (15). Higher postoperative nadir PSA levels may indicate residual cancer foci after TUEP (13). However, complete removal of the prostate adenoma by TUEP or “complete TURP” is mandatory to use the nadir PSA level as a biomarker of oncological outcomes, and the nadir PSA level after incomplete TUEP may not be a reliable marker. Compared with conventional TURP, TUEP is more technically demanding and may pose a challenge for urologists-in-training. In our cohort, 22 (5.1%) of the 428 men who underwent TUEP were excluded because of incomplete adenoma removal (<70%). Up to 94.9% men underwent acceptable TUEP in terms of adenoma removal. It has been shown that the mean enucleation ratio for beginners’ hand was approximately 60% and increased to 65% after 90 patients (23, 24). This may explain why previous studies did not show a consistent nadir PSA threshold that predicts oncological outcomes.

A previous study showed that PSA declined by 81%–86% after TUEP, which correlated with the amount of removed prostate tissue (25). We noted a higher PSA reduction rate (>90%) in our cohort. Elmansy et al. showed that percentage PSA reduction was significantly lower in the malignant group than in the benign group (47% vs. 75%, respectively, p < 0.001) (13). In contrast, Otsubo et al. reported that percentage PSA reduction was similar in the benign and cancer groups (83.2% vs. 83.2%, p = 0.962) (22). The major difference between the two studies was the inclusion of patients with different zonal distributions of cancer. Elmansy et al. excluded patients with incidental PCa in TZ but included patients with PCa in the remaining prostate that was newly diagnosed by TRUSP Bx during the post-TUEP follow-up period. Patients with newly diagnosed PCa may already have cancer in PZ that was not detected during TUEP; therefore, the post-TUEP PSA level was high and percentage PSA reduction was low. In Otsubo et al.’s study, the cancer group included men with incidental PCa found in TZ. They concluded that men with incidental PCa in TZ may exhibit PSA reduction similar to that in men with benign lesions, similar to our findings.

Tumor GS may also influence the magnitude of PSA reduction and PSA nadir. Rivera et al. showed that patients with GS ≥8 cancer had a significantly higher PSA nadir than did patients with GS ≤7 (p = 0.01); this was also observed in our PZ group (26). By categorizing cancer groups with different zonal distributions, we showed a more clear association between post-TUEP nadir PSA and oncological outcomes, thus making the nadir PSA more clinically meaningful.

Otsubo et al. also reported that men with incidental PCa had higher postoperative PSAV than men in the benign group after TUEP (0.22 vs. 0.04 ng/ml/year, p = 0.003); we believe this may largely relate to residual cancer nests in the remaining prostate (22). Elmansy et al. showed that PSAV was significantly higher in the late cancer group than in the benign group (13). Our results confirmed the findings of Elmansy et al. by showing that TTPP was longer in the benign group than in the three cancer groups.

About 78% incidental PCa lesions found in TUEP are reportedly indolent cancers with GS ≤6 (21). Although the majority of these cancers can be managed by active surveillance, the optimal treatment strategy for incidental PCa remains controversial (21, 26, 27). Interestingly, 2%–47.8% patients with incidental PCa showed no residual cancer after radical prostatectomy (pT0); this indicated that TUEP or TURP may have removed all cancer nests in these patients (20). In contrast, 5.6% patients showed extracapsular extension (pT3), and 31% showed upgraded GS (4, 12). In this context, the postoperative PSA nadir may be used to detect significant residual cancer after TUEP.

The prostate volume measured by TRUS is likely to underestimate the prostate weight (28). The inherent error rate varied with the prostate size. Prostate glands between 60 and 80 g had the best-calculated accuracy by TRUS (28). Rodriguez et al. reported that prostate volume measured by displaced water volume had a correlation coefficient of 0.997 with the prostate weight, indicating that the specific density of the prostate tissue is roughly 1.003 (29). Thus, it is not surprising that enucleated adenoma percentage was sometimes higher than 100% due to the volume and weight relationship.

Our study had several limitations. First, this was a retrospective study in which postoperative PSA levels were not measured at predefined time points; thus, the nadir PSA levels were not precisely determined. Second, cancer phenotypes may not represent the true spectrum of patients in this setting. Selection bias may exist when making TUEP-related decisions. However, if we categorize patients with cancer according to the anatomical zonal distribution, PSA kinetics are still reliable and useful in clinical applications. Third, only 63.3% of patients with suspected PCa underwent perioperative TRUSP Bx, which may have led to the underestimation of PZ cancer. Among 70 men who eventually presented with PSA ≧1.7 ng/ml, 16/70 (22.9%) men were re-biopsied following the surgery; 22/70 (31.4%) men were followed by MRI. In addition, five men without elevated PSA postoperatively were re-biopsied and followed by MRI for regular active surveillance protocol. Fourth, we adopted a cutoff value of ≥70% in the definition of complete adenoma removal according to prior studies (23, 24). The ideal criterion to define complete TUEP remains unclear.

After complete TUEP, nadir PSA ≥1.7 ng/ml may inform the presence of residual cancer and/or cancer foci with GS ≥7 in the remaining prostate. For men with incidental PCa found in the removed adenoma and a nadir PSA of <1.7 ng/ml, active surveillance or other conservative management may be preferred over active treatments. TUEP may generate a new baseline PSA to monitor PCa progression. Whether progressive PSA elevation from below to above 1.7 ng/ml after TUEP indicates recurrence of GS ≥7 cancer nests needs further investigation with a longer follow-up duration.

## Data Availability Statement

The raw data supporting the conclusions of this article will be made available by the authors, without undue reservation.

## Ethics Statement

The studies involving human participants were reviewed and approved by Research Ethics Committee Office of National Taiwan University Hospital (202009014RINA). Written informed consent for participation was not required for this study in accordance with the national legislation and the institutional requirements.

## Author Contributions

Study concept and design: Y-TC, Y-SP, and C-HC; Acquisition of data: Y-TC, J-HH, Y-CL, Y-KC, S-CH, K-KF, S-PL, P-MC, H-CC, and Y-SP; Data and statistical analysis: Y-TC, J-HH, Y-SP, and C-HC; Manuscript editing: Y-TC and Y-SP; Critical review of the manuscript: Y-SP, C-HC, and J-HH; All authors read and approved the final manuscript.

## Conflict of Interest

The authors declare that the research was conducted in the absence of any commercial or financial relationships that could be construed as a potential conflict of interest.

## Publisher’s Note

All claims expressed in this article are solely those of the authors and do not necessarily represent those of their affiliated organizations, or those of the publisher, the editors and the reviewers. Any product that may be evaluated in this article, or claim that may be made by its manufacturer, is not guaranteed or endorsed by the publisher.

## Abbreviations

AUC, area under the curve; BPH, Benign prostatic hyperplasia; CZ, central zone; GS, Gleason score; PCa, prostate cancer; PSA, prostate-specific antigen; PSAV, postoperative PSA velocity; PSAW, PSA reduction per gram of enucleated prostate weight; PZ, peripheral zone; TRUSP, transrectal ultrasound of the prostate; TRUSP Bx, TRUSP-guided biopsy; TTPP, time to PSA progression; TUEP, Transurethral enucleation of the prostate; TURP, Transurethral resection of the prostate; TZ, transition zone; ROC-CA, receiver operating characteristic curve analysis.
